# Assessing the Efficiency of Triangular Gold Nanoparticles as NIR Photothermal Agents In Vitro and Melanoma Tumor Model

**DOI:** 10.3390/ijms232213724

**Published:** 2022-11-08

**Authors:** Sorina Suarasan, Andreea Campu, Adriana Vulpoi, Manuela Banciu, Simion Astilean

**Affiliations:** 1 Nanobiophotonics and Laser Microspectroscopy Center, Interdisciplinary Research Institute in Bio-Nano-Sciences, Babes-Bolyai University, 42 T. Laurian Str., 400271 Cluj-Napoca, Romania; 2 Nanostructured Materials and Bio-Nano-Interfaces Center, Interdisciplinary Research Institute in Bio-Nano-Sciences, Babes-Bolyai University, 42 T. Laurian Str., 400271 Cluj-Napoca, Romania; 3 Center of Systems Biology, Biodiversity and Bioresources, Department of Molecular Biology and Biotechnology, Faculty of Biology and Geology, Babes-Bolyai University, 5-7 Clinicilor Str., 400006 Cluj-Napoca, Romania; 4 Department of Biomolecular Physics, Faculty of Physics, Babes-Bolyai University, 1 M. Kogalniceanu Str., 400084 Cluj-Napoca, Romania

**Keywords:** triangular gold nanoparticles, photothermal agents, photothermal therapy, biological phantoms, melanoma cells

## Abstract

Photothermal therapy (PTT) is gaining a lot of interest as a cancer treatment option with minimal side effects due to the efficient photothermal agents employed. They are based on nanomaterials that, upon laser irradiation, absorb photon energy and convert it into heat to induce hyperthermia, which destroys the cancer cells. Here, the unique light-to-heat conversion features of three different gold nanotriangular nanoparticles (AuNTs) are evaluated with respect to their absorption properties to select the most efficient nanoheater with the highest potential to operate as an efficient photothermal agent. AuNTs with LSPR response in- and out- of resonance with the 785 nm near-infrared (NIR) excitation wavelength are investigated. Upon 15 min laser exposure, the AuNTs that exhibit a plasmonic response in resonance with the 785 nm laser line show the highest photothermal conversion efficacy of 80%, which correlates with a temperature increase of 22 °C. These photothermal properties are well-preserved in agarose-based skin biological phantoms that mimic the melanoma tumoral tissue and surrounding healthy tissue. Finally, in vitro studies on B16.F10 melanoma cells prove by fluorescence staining and MTT assay that the highest phototoxic effect after NIR laser exposure is induced by AuNTs with LSPR response in resonance with the employed laser line, thus demonstrating their potential implementation as efficient photothermal agents in PTT.

## 1. Introduction

Cancer treatment options depend on the cancer type and stage, but typically it involves a combination of surgery, chemotherapy, and radiotherapy. Nonetheless, these therapeutic strategies are not suitable for every patient and have a great variety of possible side effects, such as drug resistance or damage to healthy tissues [1,2]. A new cancer treatment strategy attracting more and more interest is the photothermal therapy (PTT) [3,4,5]. The approach is based on the conversion of light energy into heat to induce hyperthermia and, implicitly, destroy cancer cells. When combined with gold nanoparticles (AuNPs) as photothermal agents, PTT can be highly efficient proving minimal side effects [6,7,8,9]. Specifically, in the tumoral tissue, which is characterized by blood vessels with discontinuous capillary walls and a dysfunctional lymphatic system, AuNPs with specific optical and chemical properties can penetrate and accumulate effectively. Upon laser exposure, AuNPs absorb the photon energy and convert it into heat, which dissipates and induces an increase in the local temperature around AuNPs and, therefore, damage to the cancer cells by hyperthermia [10,11].

Usually, for PTT applications, lasers with wavelengths and AuNPs with localized surface plasmon resonance (LSPR) in the near-infrared (NIR) region are highly desirable. This region is the so-called biological window, where the absorption and scattering of hemoglobin and water are reduced [12]. In this tissue’s transparent bio window, the laser beam can penetrate deeper into tissues without damaging the surrounding healthy cells and beneficially stimulate the accumulated AuNPs. However, to be efficient photothermal agents, AuNPs are required to have a size between 10 and 100 nm, LSPR band in the NIR region (650–900 nm), to present high photothermal conversion efficacy, and high biocompatibility and stability in physiological conditions [13,14]. So far, differently-shaped AuNPs were tested as photothermal agents [15,16,17,18]. For example, Gao et al. [19] employed multilayered gold nanoshells to kill up to 90% of carcinoma cells, which were irradiated for 5 min with an 808 nm laser line. Knights et al. [20] used targeted gold nanorods (AuNRs) with LSPR at 850 nm to study the effect of continuous and pulsed 854 nm lasers for PTT of lung cancer cells. Ma et al. [13] used gold nanoprisms to apply PTT on breast cancer cells at low laser power density (2 W/cm^2^) and Ren et al. [21] conducted a theoretical and experimental study to assess the properties of AuNRs compared to gold nanotriangles (AuNTs). They concluded that the overall photothermal conversion efficiency of AuNTs is higher, demonstrating their superiority as photothermal agents. Boca et al. [22] also proved that silver NTs induce a higher cell mortality rate under NIR laser excitation compared to AuNRs.

Therefore, among the different AuNPs exploited as thermoplasmonic generators in PTT, we selected for this study AuNTs due to their unique tunable LSPR response and effective photothermal properties. AuNTs present two LSPR bands, one located in the visible region attributed to out-of-plane resonance and a second band in NIR region due to the in-plane resonance, which is highly dependent on the edge length of AuNTs and usually presents an absorption maximum in the 900–1200 nm range, limiting their application [23]. To overcome this inconvenience and to shift the LSPR band of AuNTs to the optimal range for lasers and PTT, we use gelatin biosynthesized AuNTs. This type of AuNTs exhibits a tunable in-plane resonance in the 650–1200 nm range as a function of reacted gelatin concentration during the synthesis. Additionally, the gelatin covering confers also high biocompatibility and stability to AuNTs [24,25] and can act as a drug delivery matrix [26,27,28] to assist the further development of theranostic agents [29,30,31]. It must be pointed out, that due to the “bio” character of the synthesis, realized in the absence of any strong reducing agents, AuNTs are synthesized together with gold nanospheres (AuNSs), but as shown later in this study, they do not influence the PTT effect of AuNTs.

First, we synthesized AuNTs with LSPR bands centered at 690, 780, and 890 nm, denoted as AuNTs@690, AuNTs@780, and AuNTs@890, respectively. They were characterized and investigated as potential photothermal agents under a continuous 785 nm laser irradiation both in colloidal solution and biological phantoms, that mimic the biological tissue. We found that differently-sized AuNTs with plasmonic responses in- and out- of laser resonance have different absorption efficiencies and, therefore, different photothermal conversion performances. AuNTs@780 present the highest temperature increase of around 22 °C under laser irradiation, proving that the best photothermal conversion efficacy is achieved for AuNTs in resonance with the excitation laser line. To demonstrate this result, we performed in vitro studies on melanoma cells, the most lethal type of skin cancer that often gives metastases. Specifically, the cells were first treated with AuNTs for 24 h to allow the NPs internalization and then exposed to NIR irradiation for 15 min. The evaluation of viable and dead cells was assessed after irradiation by MTT assay and fluorescence imaging using viability fluorescence staining with calcein-AM and propidium iodide (PI).

The novelty of our study is represented by the evaluation of the PTT performance of AuNTs inside skin-like biological phantoms that mimic the melanoma AuNTs treated tissue and surrounding healthy tissue upon NIR irradiation. Inside the biological phantoms, under laser excitation, AuNTs from the “tumoral area” show a fast temperature increase capable to induce hyperthermia in the desired region. The heat dissipation to the nearby tissue is minimal, suggesting that the healthy cells will not be affected by localized PTT treatment. It is worth mentioning that these effects are preserved on different colored skin-like phantoms, hence the proposed PTT treatment is suitable for patients regardless of their skin color.

Therefore, in terms of PTT therapy, this study brings us a step forward to the understanding of the PTT effect on skin-like biological phantoms that could facilitate the AuNTs translation into clinical studies.

## 2. Results and Discussion

### 2.1. Gold Nanotriangles Characterization

**Figure 1:**
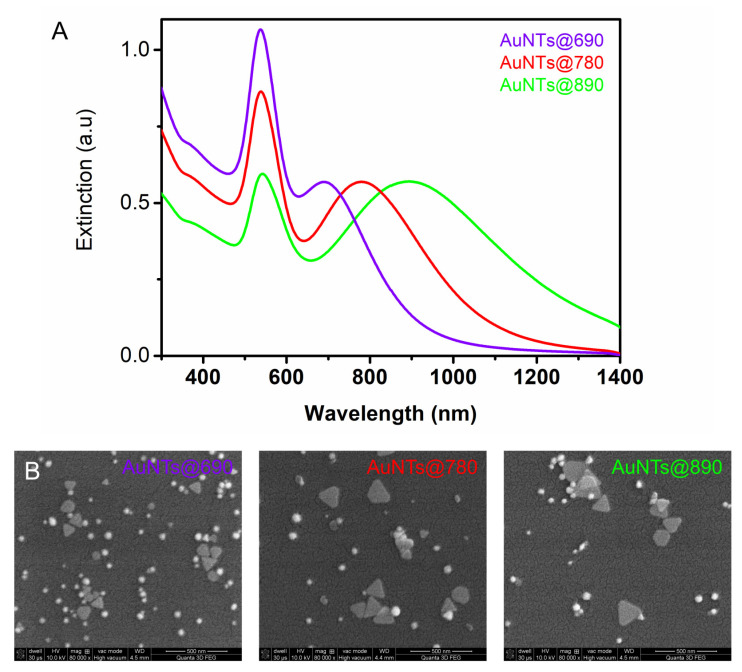
(**A**) LSPR spectra of gelatin coated AuNTs (**B**) SEM images of AuNTs. The scale bars represent 500 nm.

The synthesized colloidal solutions were first investigated using a UV-Vis-NIR spectrophotometer. The recorded extinction spectra in Figure 1A show the LSPR response of AuNTs that exhibit two well-defined bands. The first band is assigned to the out-of-plane dipole resonance of triangular AuNPs, while the second band corresponds to the in-plane dipole resonance. Considering the “bio” character of our synthesis method that employs gelatin as the only reducing and stabilizing agent, it is difficult to obtain a pure colloidal AuNTs solution uncontaminated by nanospheres. Therefore, as seen in the SEM images presented in Figure 1B, apart from AuNTs, spherical byproducts are obtained in all three samples. The plasmon resonance band of co-synthesized AuNSs overlaps with the out-of-plane dipole resonance of AuNTs centered at 534 nm. As we demonstrated in our previous work [24], the in-plane band of AuNTs evolves in time as a function of gelatin concentration. Herein, we selected an appropriate gelatin concentration, that allows the formation of differently-sized AuNTs with the in-plane resonance bands centered at 690, 780, and 890 nm. These AuNTs with specific LSPR bands in the NIR region were selected to be in-resonance and out-of-resonance with the 785 nm laser line, in view of their future investigation as photothermal agents. It is well known that for photothermal therapeutic applications, the NIR region has great potential and is highly desired due to the optical transparency of tissues in this “biological window”, where light can deeply penetrate into the tissue. It must be noted that AuNSs do not significantly influence the results obtained from AuNTs, as we will show later, therefore, in the next studies, we will refer only to the AuNTs.

The SEM images of AuNTs, presented in Figure 1B, confirm the synthesis of different sized AuNTs as a function of the gelatin concentration present in the reaction. The shift of the in-plane dipole resonance of AuNTs from 890 to 690 nm is correlated with a decrease in AuNTs edge from 155 to 95 nm, as the SEM image analysis shows. Moreover, an increase in AuNSs concentration with increasing gelatin concentration is noticed. The surface charge of AuNTs was also investigated and found to be similar for all AuNTs (+25–30 mV) due to their gelatin coating.

### 2.2. Investigation of the AuNTs Light-to-Heat Conversion Performances in Solution

**Figure 2:**
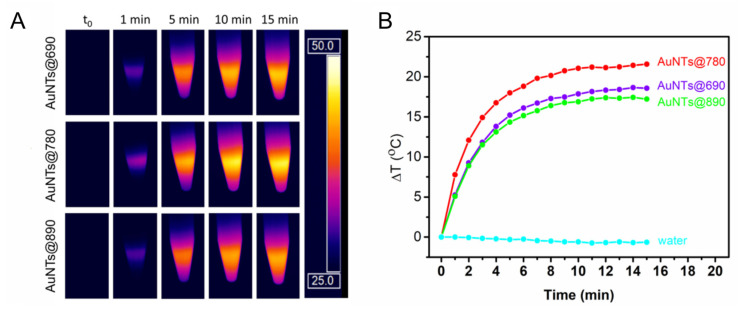
(**A**) The thermographic images of AuNTs during the irradiation with a 785 nm laser for 15 min and (**B**) The extracted corresponding thermal curves.

To evaluate the efficiency of AuNTs as photothermal agents in aqueous solutions, the light-to-heat conversion performances of the synthesized NPs were investigated by exposing the colloidal solutions to a 785 nm laser line for 15 min. During the irradiation, thermographic images were recorded by an infrared thermal camera (Figure 2A). From the recorded thermographic images, the temperatures (T) were extracted and the temperature difference (Δ*T*) was calculated with regard to the temperature of the environment (T_amb_). Next, Δ*T* were plotted relative to the irradiation time to obtain the thermal curves for all colloidal AuNTs solutions. As displayed in Figure 2B, a rapid increase in the temperature of all irradiated AuNTs is noticed in the first 10 min that slightly continues to rise until 15 min when the irradiation is stopped. As depicted by the thermal curves, 15 min of irradiation are sufficient to reach the highest generated temperature, since the temperature increase is rather small after 10 min, the steady-state temperature being reached. All AuNTs were demonstrated to convert light-to-heat; however, they exhibit different performances. The AuNTs with LSPR bands out-of-resonance with the excitation wavelength show an increase in the temperature of 19 °C for AuNTs@690 and 17 °C for AuNTs@890 reaching temperatures of 44 and 43 °C, respectively. As expected, the AuNTs@780 shows the best conversion performance, reaching a temperature of 47 °C by increasing the T_amb_ with 22 °C. The AuNTs in resonance with the laser line present better absorption properties at the 785 nm wavelength, thus being able to generate heat more efficiently. An equivalent volume of water irradiated at the same experimental parameters was used as control, to exclude any thermal contributions from the solvent.

The temperature increases recorded for AuNTs are more than enough for PTT therapy, which requires only a 5–10 °C temperature increase near the tumoral tissue for efficient apoptosis of the tumoral cells.

A AuNSs@534 sample was also investigated as a control to assess the contribution of the co-synthesized gold nanospheres from the colloidal AuNTs solutions. Figure S1 in Supplementary Materials proves that AuNSs do not significantly increase the temperature under 785 nm laser irradiation, since they have the LSPR band far from the laser resonance condition. As it can be seen, after 15 min irradiation, they present only a 3.87 °C temperature increase (Figure S1).

**Figure 3:**
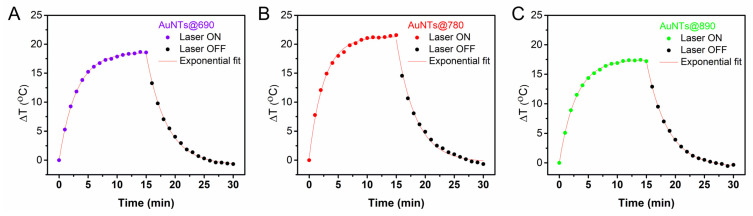
The thermal curves of the AuNTs with LSPR at (**A**) 690, (**B**) 780, and (**C**) 890 nm, respectively.

Further, the photothermal conversion efficiencies (η) were calculated using Equation (2), considering both the heating and cooling behavior of each sample (Figure 3). Hence, η of 73% was determined for AuNTs@690, 64% for AuNTs@890 and 80% for AuNTs@780, while only 12% efficiency was obtained for AuNSs@534. These results are in good agreement with the previous observations. AuNTs@780 present the highest potential to be further implemented as photothermal agents, whereas the co-synthesized AuNSs do not exhibit a significant thermal contribution, thus their separation is not mandatory.

Also, we tested the effect of the AuNTs@780 concentration on the temperature increase under laser irradiation. The optical density (OD) of AuNTs tested throughout all investigations was 0.56. For this study, we also tested AuNTs@780 with an OD of 1.12 and 0.28, respectively. As can be seen in Figure S2 in Supplementary Materials, the behavior of different concentrations of AuNTs@780 is similar, showing a correlation between OD and temperature increase. The maximum temperature reached is almost identical for AuNTs@780 with an OD of 0.56 and 1.12 and slightly lower for AuNTs@780 with a lower OD of 0.28. This result is consistent with the trend observed previously by Campu et al. [32], who concluded that a higher OD of AuNPs under irradiation does not significantly increase the reached maximum temperature, but the heating process is faster.

### 2.3. Investigation of the AuNTs Light-to-Heat Conversion Performances in Biological Phantoms

Next, the AuNTs photothermal performances were investigated in biological phantoms in order to assess their photothermal activity in simulated tissue for further implementation in in vitro PTT applications. These phantoms are tissue-mimicking models designed to simulate specific properties of the human body, such as light propagation and scattering, or electrical conductivity. The biological phantoms investigated in this study are based on agarose. They are semi-solid hydrogel structures made from agarose, water, and intralipid as a scatterer, that mimic the human skin [33]. In the case of photothermal agents, biological phantoms are essential as an intermediate stage for medical application to allow the evaluation of AuNPs effects on simulated tissue. This evaluation gives the possibility of AuNPs dose, laser intensity, and irradiation time adjustment to optimal values capable to induce the local temperature increase and subsequent apoptosis of cancer cells under irradiation.

**Figure 4:**
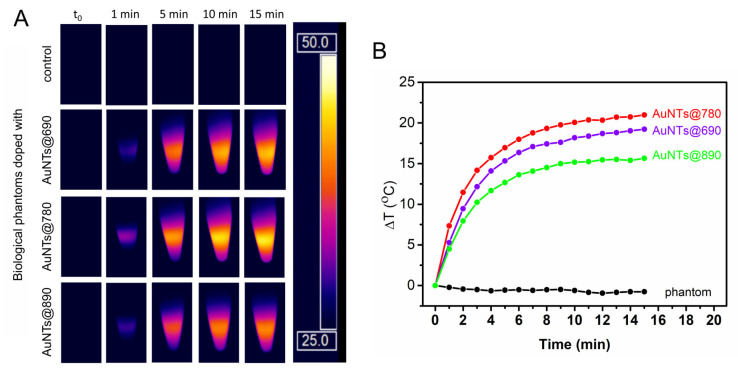
(**A**) The thermographic images of AuNTs doped biological phantoms during the irradiation with a 785 nm laser for 15 min and (**B**) the extracted corresponding thermal curves.

The phantoms developed here were doped with AuNTs having different optical properties, at the same final concentration as in the solution. As can be seen in Figure 4, under the laser irradiation, the temperature of the control biological phantom remains constant during the experiment, while, as expected, the temperature of the phantoms doped with AuNTs increases. The doped phantoms show a temperature profile influenced by the LSPR response of the AuNTs, revealing a behavior similar to the one observed in the case of the colloidal AuNTs. Specifically, biological phantoms doped with AuNTs@780 present the highest increase in Δ*T* (21 °C), followed by the phantoms doped with AuNTs@690 and AuNTs@890 as determined based on the thermal maps recorded in real-time from biological phantoms during the irradiation (Figure 4A). Therefore, we can conclude that, upon the integration in biological phantoms, AuNTs preserve their light-to-heat conversion properties as a LSPR band in resonance with the irradiating laser confers a higher absorption efficiency to the AuNTs, which convert the optical energy into thermal energy, resulting in a higher temperature increase. So, further, we will investigate only the in vitro efficacy of AuNTs@780.

It must be pointed out that considering that all cells are sensitive to temperature increase and excessive heating can lead to cellular death, for an efficient PTT treatment, the cancerous tissues irradiation should be carefully controlled to reduce the side effects on normal cells at a minimum. As we already mentioned, AuNTs are accumulating preferentially into cancer cells due to the physiological characteristics of the tumoral tissue, but thermal diffusion could appear. Another important aspect in PTT therapy is that the cellular death should be through apoptosis and not necrosis, to avoid cancer recurrence and metastasis due to inflammatory response. Therefore, it is crucial to carefully increase the temperature only in the tumoral tissues and up to a maximum of 50 °C to induce apoptosis and not necrosis, which occurs above 50 °C [34,35]. In this context, considering the ability to control their absorption properties, gold nanoparticles are excellent candidates of choice. For instance, the NIR therapeutic effect of gold bipyramids was demonstrated to be efficient in in vitro experiments, showing a massive antitumor efficiency and treatment localization limited to the irradiated area [36].

**Figure 5:**
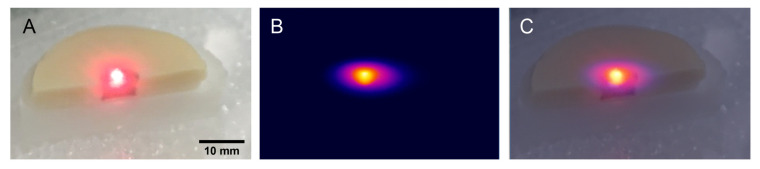
(**A**) Cross-section picture of a skin-like phantom with two different regions that mimic the normal and tumoral tissue treated with AuNTs@780, under laser irradiation, (**B**) thermal contrast image of the skin phantom after 5 min of laser irradiation, (**C**) merged A and B images.

To simulate and investigate the controlled irradiation of tumoral tissue and heat dissipation to nearby healthy cells, we used an agarose-based two-layer skin phantom with two distinct regions corresponding to healthy and tumoral tissue. The skin-like biological phantom was obtained as described in the Section 3 by attaching together a dermal and an epidermal-like layer that contains a certain amount of coffee solution and intralipid to mimic the color, absorption, and scattering properties of real tissues [37]. To simulate the tumoral tissue treated with AuNTs, in the epidermal layer of the skin phantom a puncture was made and filled with a mixture of base phantom material and AuNTs@780. Figure 5A presents a section of the skin phantom under laser irradiation, with the epidermal layer showing two different regions. One region simulates the tumoral tissue, consisting of AuNTs doped phantom, and the other one, surrounding the tumoral tissue, mimicking the normal tissue. To examine the temperature elevation, the PTT effect was triggered by a 785 nm laser irradiation of the phantom. Due to AuNTs present in the tumoral phantom, the temperature in the middle section increases up to 45.5 °C after only 5 min of laser exposure. As it can be seen in Figure 5B and 5C, the thermal contrast image shows that the temperature of the surrounding normal tissue remains constant, suggesting that the heat dissipation is minimal.

**Figure 6:**
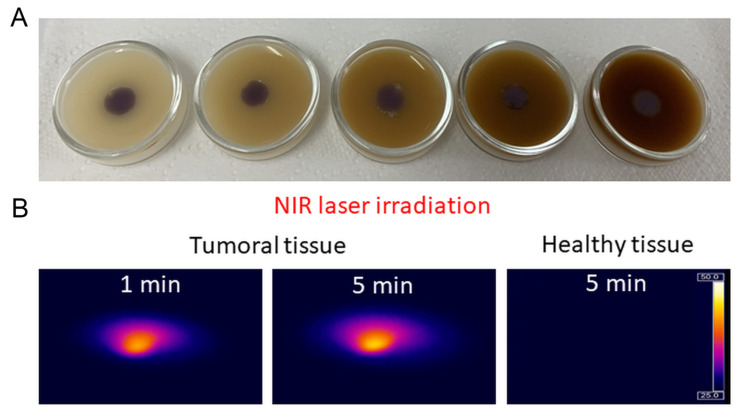
(**A**) Different skin tones biological phantoms which mimic a tumoral region and the surrounding healthy tissue. (**B**) Temperature contrast images of the NIR laser irradiated tumoral and healthy tissue phantoms.

Moreover, it is worth mentioning that the temperature of the normal mimicking tissue phantom remains unchanged under laser irradiation. Nonetheless, to test the possible influence of patient skin color on the effect of the PTT treatment, we have investigated skin phantoms that mimic the different skin color types. As it can be seen in Figure 6A, the color of the epidermal layer of the phantoms was adjusted to mimic different skin tones from pale to dark. To obtain these skin tones, we used a pigment similar to melanin, called melanoidin, which is present in coffee solution. To simulate a melanoma tumor treated with AuNTs@780, we made a puncture in the middle of each skin phantom and filled it with AuNTs@780 doped phantom material. The surrounding tissue of each tumor was considered as healthy tissue and used as control in the following PTT experiments. After all different skin phantoms were prepared, they were cooled down to 5 °C for about 30 min before laser irradiation. Under NIR laser irradiation, the temperature of the phantom tumoral tissue treated with AuNTs@780 has increased by 18 °C in just 1 min and then slowly rises with approx. 2 °C after 5 min of irradiation (Figure 6B). Considering this high-temperature increase, more than an efficient PTT requires, after 5 min the laser was turned off. It is important to note that the phantom regions mimicking the healthy tissue in all different skin phantoms remained unchanged even after 5 min irradiation. The dissipated heat from the AuNTs present in the laser irradiated tumor is minimal, increasing the temperature of the surrounding regions by 2–3 °C, which is safe for healthy tissues. However, in real clinical studies, the heat dissipation can be reduced even more by applying, for example, multiple short laser irradiation sessions or reducing the laser power.

Therefore, corroborating the data acquired from AuNTs in biological phantoms, we can conclude that AuNTs are excellent plasmonic nanogenerators for PTT and contrast agents for thermographic imaging. Moreover, since it is important to induce cellular death via apoptosis, a minimal laser irradiation period is enough to increase the temperature up to 45–50 °C, for an efficient PTT treatment.

### 2.4. Bio- and Thermostability of AuNTs@gelatin

In view of future biomedical applications of AuNTs in vitro in melanoma cells, the colloidal stability was evaluated under a simulated physiological ionic solution of 0.9% NaCl and cellular media at a physiological temperature of 37 °C. In biological environments, AuNPs could become prone to aggregation, which significantly affects their properties and internalization. In this regard, we tested the stability of our different-sized AuNTs by monitoring the plasmonic response in 0.9% NaCl solution and DMEM cellular medium at 37 °C. For exemplification, we show the data recorded only for one type of AuNTs, specifically AuNTs@780. As can be seen in Figure S3 in Supplementary Materials, no sign of aggregation is noticed in LSPR spectra of AuNTs under different physiological conditions. Moreover, in phenol-free cellular medium no shift in the LSPR band of AuNTs can be noticed, but only a minor broadening of the spectrum. This attests that proteins from the media do not attach to the surface of AuNPs. However, the spectra broadening can be attributed to the overlap of the AuNTs extinction with the light scattering of serum proteins from the medium [38].

The stability of AuNTs under laser irradiation is also an important factor for a good nanoheater in PTT applications. To investigate the thermal stability of AuNTs, they were tested in a biological phantom doped with AuNTs following 4 cycles of irradiation. Explicitly, in one cycle, the laser is switched on for 15 min to irradiate the phantom and then switched off for another 15 min until the phantom reaches the initial temperature. After each cycle, the maximum temperature reached is similar, proving the thermal stability and reusability of the AuNTs under laser exposure. Here we exemplified the AuNTs stability using the AuNTs@780 sample (Figure S4 in Supplementary Materials). Therefore, we conclude that the photothermal effect of AuNTs illustrated in Figure S4 is stable and repeatable for at least four irradiation cycles, indicating that the phototherapy could be repeated if necessary without significant heat loss.

### 2.5. In Vitro NIR Phototherapy

Further, we were interested to evaluate and compare the phototherapeutic effect of each AuNTs in vitro under NIR laser irradiation. In this context, we used a B16.F10 murine melanoma model. Specifically, the cells were first incubated with different AuNTs under the same conditions for 24 h. After treatment, the cells were washed with PBS to remove the uninternalized AuNPs and irradiated for 15 min with a 785 nm laser line using a power of 196 mW and a laser spot of about 1.5 mm. To assess the in vitro PTT effect on the cells, two different experiments were performed: fluorescence imaging and MTT assay.

**Figure 7:**
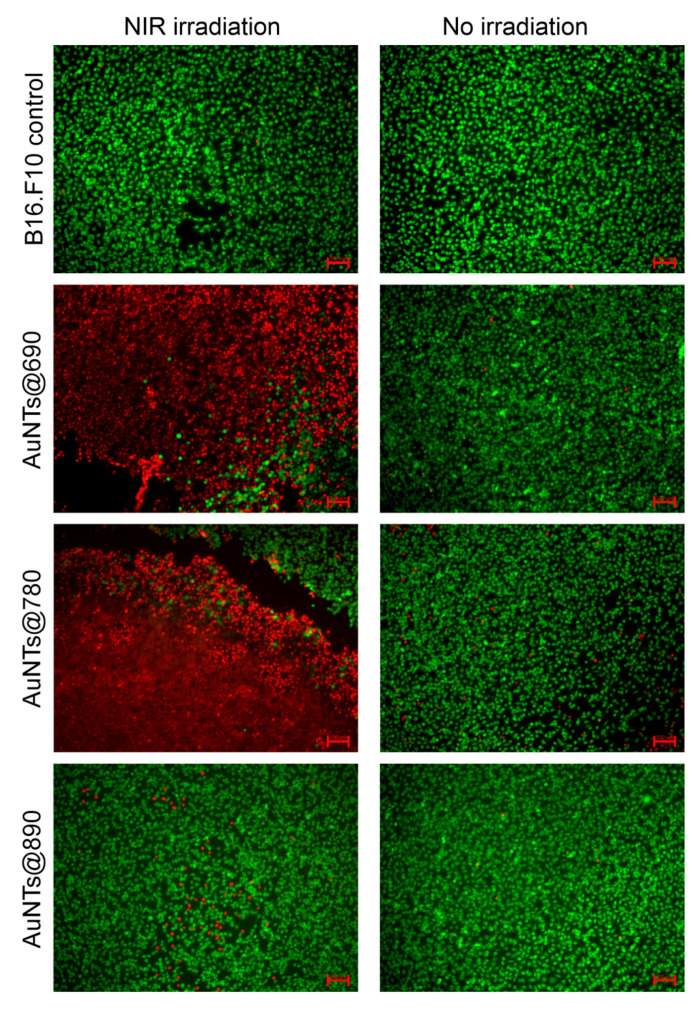
Merged fluorescence images of B16.F10 cells stained with calcein-AM and PI before and after NIR irradiation. The scale bars represent 100 μm.

In the first experiment, immediately after irradiation, the cells were stained with calcein-AM and propidium iodide, fluorophores commonly used to label viable and dead cells. Cells treated with AuNSs@534, untreated cells, and cells treated with different AuNTs, but not irradiated, were used as control groups. Fluorescence staining images of NIR irradiated and non-irradiated cells are presented in Figure 7. As it can be seen, the untreated cells are not affected by the laser irradiation, suggesting that the laser alone is unable to induce any therapeutic effect. However, the cells previously incubated with AuNTs and irradiated present viability dependent on the AuNTs treatment type received. For example, the irradiation of cells treated with AuNTs@890 does not induce considerable cell death, only a few cells being labeled with PI compared to the non-irradiated sample. However, a powerful effect of the same irradiation period can be noticed in the case of the cells treated with AuNTs@690 and AuNTs@780. AuNTs@780 presents the best therapeutic effect, as proved by the high number of dead cells in the irradiated area. Compared to the irradiated cells, the non-irradiated cells are mostly stained by calcein-AM, proving that the incubation with different AuNTs does not highly impact the cell viability. This significant difference between the in vitro therapeutic efficiency of the investigated AuNTs is related to the different resonances and size of AuNPs, which impact their light-to-heat conversion, as we shown above, and efficient internalization in time. We hypothesize that the reduced PTT effect in the case of AuNTs@890 treatment is due to a combination of two factors: the increased size of AuNTs that are not efficiently internalized in the cells and the LSPR response far from the resonance condition of the agglomerated AuNTs internalized, which do not efficiently absorb the laser energy. To prove again that the influence of the gold nanospheres co-synthesized with AuNTs is minimal, a control experiment was conducted. The double staining fluorescence images of AuNSs@534 treated cells (Figure S5 in Supplementary Materials) show that under NIR laser irradiation only a reduced number of cells are affected, proving no significant effect.

**Figure 8:**
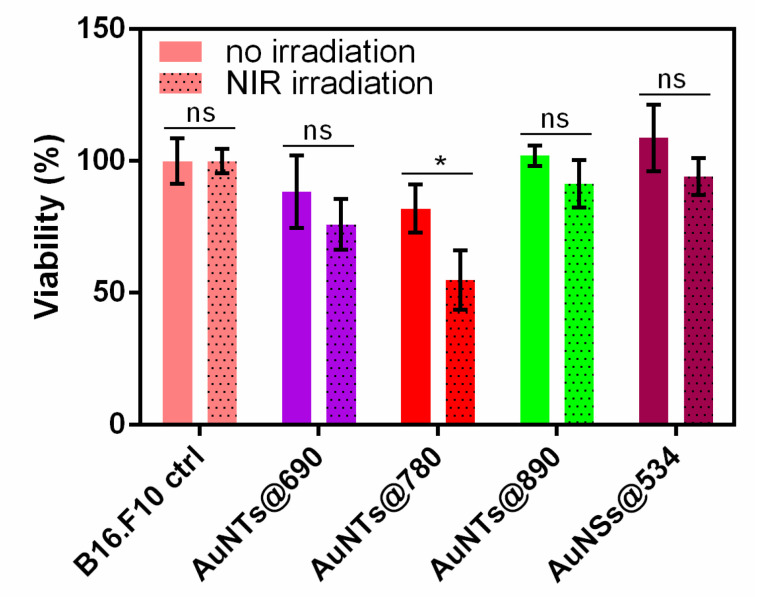
Cell viability of B16.F10 cells treated with different AuNPs before and after NIR irradiation. Two-way ANOVA analysis with Bonferroni posttest (ns, not significant, *p* > 0.05; * *p* < 0.05).

To further confirm the PTT effect of AuNTs internalized in B16.F10 cells, we monitored the cellular viability after NIR irradiation by employing the MTT assay. The test was performed under similar conditions as for the fluorescence staining assay, but a tetrazolium dye was used to evaluate the metabolic activity of the viable cells. As shown in Figure 8, cell viability assessed by this colorimetric assay confirms that all AuNTs exhibit phototherapeutic activity against melanoma cells under NIR laser irradiation. A decrease in cell viability to 88 and 82% is noticed for the cells treated with AuNTs@690 and AuNTs@780, compared to the control cells. After irradiation, the untreated cells viability is unaffected, while the viability of cells treated with AuNTs@690 and AuNTs@780 decreases even more to 76 and 55%, suggesting a strong phototoxicity of the AuNTs@780. However, in the case of the cells treated with AuNTs@890, the cell viability remains quite high (91%) even after laser irradiation. This could be due to the increased size of AuNTs that are not efficiently uptaken by the cancer cells during the incubation period and to the internalized AuNTs LSPR far out-of-resonance of irradiating laser. On the other hand, cells treated with AuNSs@534 are mostly unaffected after irradiation, confirming once again the negligible effect of the nanospherical impurities from the AuNTs samples on the phototherapeutic effect of AuNTs. Quite the opposite, as we previously demonstrated [24,39], the cells treated only with AuNSs present a slightly improved growth and proliferation rate compared with the control untreated cells. To better evidence the NIR irradiation effect on the cells treated with different AuNTs, statistical analysis was performed on each cell group before and after laser irradiation. *p*-Value was determined to evaluate the statistical significance of the data and was calculated by Two-way ANOVA analysis with Bonferroni correction. As it can be seen in Figure 8, the laser irradiation has a significant result in the case of AuNTs@780 treated cells.

Therefore, the MTT data confirm the fluorescence staining results by revealing a more pronounced phototoxicity for the AuNTs@780 sample that induces a higher cellular mortality rate after 15 min irradiation in the same conditions as for the rest of the treated cells.

## 3. Experimental Section

### 3.1. Materials

Hydrogen tetrachloroaurate-(III) trihydrate (HAuCl_4_·3H_2_O, 99.99%), gelatin type A, sodium chloride (NaCl), phosphate buffered saline (PBS) solution, 3-(4,5-dimethylthiazol-2-yl)-2,5-diphenyltetrazolium bromide (MTT) solution, Dimethyl sulfoxide (DMSO), trypsin, and propidium iodide (PI) solution were purchased from Sigma-Aldrich (Darmstadt, Germany). Dulbecco’s Modified Eagle medium, penicillin, streptomycin, L-glutamine, and fetal bovine serum (FBS) were purchased from Lonza (Basel, Switzerland). Calcein-AM was purchased from Life Technologies (Bleiswijk, Netherlands). Milli-Q water (resistivity ~18.2 MΩ cm) was used as a solvent.

### 3.2. Sample Preparation

Colloidal gold nanotriangular nanoparticles (AuNTs) with different absorption properties were bio-synthesized in the presence of a gelatin biopolymer using a previously described one-step method [24]. Briefly, in three vials, different concentrations (0.95%, 0.85%, and 0.75%) of a gelatin solution were mixed with 3 mM HAuCl_4_ solution (volume ratio 1:1) and incubated for 6 h at 80 °C to obtain gelatin-coated AuNTs, that exhibit the desired in-plane localized surface plasmon resonance (LSPR) at 690, 780, and 890 nm, respectively. Following the synthesis, the unreacted gelatin was removed from the colloidal solution by centrifugation at 6000 rpm for 5 min, discarding the supernatant and collecting the nanoparticles in Milli-Q water. The stability of the AuNTs was tested in a physiological ionic solution of 0.9% NaCl and phenol-free cell media at the physiological temperature of 37 °C. For the thermal investigations, the optical densities of the in-plane LSPR band of all synthesized AuNTs were adjusted to the same value of 0.56 a.u. Gold nanospheres (AuNSs@534) synthesized following the same procedure in the presence of a high gelatin concentration (3%) were used as a control.

The agarose tissue-like phantoms were prepared using 400 mg agarose solubilized in 10 mL hot Milli-Q water under vigorous magnetic stirring and mixed with 1 mL of intralipid until a homogeneous solution was obtained. The as-prepared biological phantoms were doped with AuNTs (AuNTs@690, AuNTs@780, and AuNTs@890) and AuNSs (AuNSs@534) at the same final concentration as in colloidal solutions investigations. The doped still liquid phantoms were carefully poured into Eppendorf tubes and maintained at room temperature for 30 min or until a semi-solid mass was formed. Until used, the phantoms were stored at 4 °C in the dark.

To test the possible influence of patient skin color on the PTT treatment effect, skin phantoms that mimic different skin color types were employed. For this purpose, two-layer skin phantoms were prepared using a modified protocol previously reported by Mustari et al. [40]. Briefly, to obtain the skin phantom, a dermal- and an epidermal-like layer were attached together. For the dermal layer of the skin, 100 mg agarose powder was solubilized in 10 mL 0.9% NaCl solution under stirring at 80–90 °C, mixed with 1 mL intralipid, and poured into a 40 mm glass petri dish to form an 8 mm thick layer. For the epidermal layer of the skin, a homogeneous mixture of agarose, intralipid, and coffee solution was poured on the cooled dermal layer to form a 4 mm thick layer. The coffee solution was used to mimic the melanin pigment of the skin, since it is known to contain a similar pigment, melanoidin [41]. By adding different concentrations of coffee, we obtained skin phantoms that mimic different skin tones from light to dark.

After the skin phantoms were prepared, a 1.25 mm puncture was made in the epidermal layer and filled with a mixture of base phantom material and AuNTs@780 to mimic the tumoral tissue of the skin. For a better visualization of the temperature increase inside the tumoral tissue-like region of the phantoms, the thickness of the epidermal and dermal layers was purposely increased to approximately 4 and 8 mm, respectively.

### 3.3. In Vitro Cellular Studies

B16.F10 murine melanoma cells (ATCC, CRL-6475) were cultured in Dulbecco’s Modified Eagle medium (DMEM), supplemented with 10% heat-inactivated fetal bovine serum (FBS), 100 IU/mL penicillin, 100 μg/mL streptomycin, and 4 mM L-Glutamine. Cancer cells were maintained as a monolayer at 37 °C in a 5% CO_2_ humidified atmosphere.

For fluorescence imaging experiments, cells were harvested via trypsinization, and 2 × 10^4^ cells/well were further grown on 24-well plates for 24 h, while for cell viability experiments 1 × 10^4^ cells/well were grown on 96-well plates for 24 h in a controlled atmosphere. After 24 h of cultivation, cells were treated with AuNTs and AuNSs colloidal solutions and additionally incubated for 24 h. Untreated cells were used as a control.

Statistical Analysis: Data from three different experiments were reported as mean value ± standard deviation (SD). To quantify the therapeutic effect of different NIR irradiated AuNTs on B16.F10 cells, a two-way ANOVA with Bonferroni correction for multiple comparisons was used. All statistical analyses were performed using GraphPad Prism 6 (Dotmatics, San Diego, CA, USA).

### 3.4. Photothermal Efficiency in Solution and Biological Phantoms

This experiment was designed to acquire the thermal curves and to visualize the distribution/dissipation of heat over the sample area. Colloidal AuNTs and biological phantoms doped with AuNTs were placed in Eppendorf tubes on a specifically designed stand that allows simultaneous laser irradiation and real-time temperature measurement at a 90° angle. Specifically, a 785 nm continuous laser line was positioned on top of the samples to allow vertical irradiation from above, while the temperature was recorded by a thermographic camera placed perpendicular to the sample. The laser power was set at 196 mW with a laser spot diameter of 3 mm and laser power density of 2.7 W/cm^2^. The camera was set to record the thermographic images every minute during the 15 min irradiation. From the recorded thermal images, the temperatures (T) were extracted in order to calculate the temperature difference (Δ*T*) with regard to the ambient temperature (T_amb_). The thermal curves were obtained by plotting Δ*T* as a function of time following an exponential fit, which is in accordance with the analytical determination of Δ*T* as [42]:

(1) ΔT=τ[I(1−10−Aλ)η+Q0∑ miCi](1−e−tτ)

where *τ* is the time constant describing the cooling process, *I* stands for the incident laser power, *A_λ_* is the correction factor of the absorption properties at the excitation wavelength, *Q_0_* represents the input energy, *m_i_* stands for the mass of each involved component, herein water as the solvent and the Eppendorf vial, *C_i_* their corresponding specific heat, and *t* is time.

Furthermore, for the above-mentioned equation, the photothermal efficiency (η) can be defined as [32]:

(2) $$\eta =\frac{h*A\Delta T-I\zeta }{I\left(1-\zeta \right)\left(1-10^{-A_{\lambda }}\right)}$$

where *ζ* is the energy fraction absorbed by Eppendorf tub and solvent, *h* represents the heat transfer coefficient and *A* stands for the area cross section of the laser exposed surface.

Based on Equation (2), η for all colloidal AuNTs were calculated and compared to assess the most efficient AuNTs for the 785 nm laser excitation.

### 3.5. Cellular Imaging Assay

For the fluorescence imaging experiments, cells grown on 24-well plates were treated with AuNTs@690, AuNTs@780, AuNTs@890, and AuNSs@534, respectively. After 24 h incubation with treatment AuNPs, the cells were washed with PBS to remove any uninternalized AuNPs and kept in a fresh medium. Prior to laser exposure, the medium was discarded and replaced with PBS. A 785 nm laser line was used to vertically irradiate the cells for 15 min at a power density of 2.7 W/cm^2^. Afterward, the cells were stained with calcein-AM and propidium iodide (PI) and fluorescence microscopy was employed to differentiate between viable and dead cells. The cells treated with AuNSs@534, untreated cells, and cells treated with different AuNTs which were not exposed to the laser line were used as control groups.

### 3.6. In Vitro Cell Viability Study

To investigate the viability of AuNPs treated B16.F10 cells following NIR laser irradiation, the colorimetric MTT assay was employed to evaluate the cell metabolic activity. For this study, cells seeded in 96-well plates were treated for 24 h with AuNTs@690, AuNTs@780, AuNTs@890, and AuNSs@534, respectively. After treatment, the cells were washed with PBS to remove the uninternalized AuNPs and a fresh medium was added to each well. The cells incubated without AuNPs and AuNPs-treated cells but not-irradiated, were used as controls. Random wells were selected, and the culture medium was replaced by PBS prior to the laser exposure of the cells to a 785 nm laser line for 15 min. After irradiation, the PBS from irradiated wells and medium from non-irradiated wells were replaced by 100 μL 0.5 mg/mL MTT solution and placed in the incubator for 1 h to allow the yellow tetrazolium dye MTT to be reduced to purple formazan by the living cells. Then, the MTT solution was replaced by a DMSO solubilizing solution that dissolves the insoluble purple formazan product. In this way, a colored solution was obtained with an optical density (OD) correlated with the percentage of viable cells. The OD of each well was measured spectrophotometrically at 570 nm with a reference wavelength of 670 nm, using a microplate reader. To establish the reproducibility of the results, each experiment was conducted in triplicate, and the mean value with the standard deviation was reported.

### 3.7. Equipment and Characterization Methods

The extinction spectra of the colloidal AuNTs solutions were recorded at room temperature by a Jasco V-670 UV-Vis-NIR spectrophotometer (JASCO Deutschland, Pfungstadt, Germany) with 2 nm bandwidth and 1 nm spectral resolution. The stability of AuNTs in ionic solutions of 0.9% NaCl at a physiological temperature of 37 °C was monitored using a Peltier cell unit attached to the spectrometer. The SEM images of AuNTs, which were placed on a cover slip glass, were obtained using a FEI Quanta 3D microscope equipped with an EDT detector, operating in high vacuum mode, using an acceleration voltage of 10 kV. The zeta potential investigations of AuNTs were performed using a Dynamic Light Scattering (DLS) analyzer (Nano ZS90 Zetasizer, Malvern Instruments, Malvern, United Kingdom) equipped with a He-Ne laser (633 nm, 5 mW). For sample irradiation, a 785 nm diode coupled to a Raman System R3000CN spectrometer was employed. The thermographic images were recorded using an Optris PI 450 infrared camera (Optris, Berlin, Germany) equipped with an O38 standard lens. The thermal images were analyzed using the Optris PI Connect software. The fluorescence cell imaging was realized using an inverted Zeiss Axio Observer Z1 microscope (Zeiss, Oberkochen, Germany) equipped with an HXP 120 lamp for excitation, a 10× objective (Zeiss, Plan-Apochromat, NA = 0.45), and an AxioCamIcc digital camera.

## 4. Conclusions

In conclusion, we have successfully evaluated and compared the light-to-heat conversion properties of three different triangular gold nanoparticles (AuNTs@690, AuNTs@780, and AuNTs@890) with resonances in- and out- of the resonance condition with respect to the 785 nm irradiating laser. After identical irradiation conditions, we demonstrated the PTT activity of all AuNTs in solution and proved that AuNTs@780 in resonance with illumination laser present the highest photothermal conversion efficacy, that leads to a temperature increase of 22 °C under NIR irradiation. This result was also confirmed by investigations performed on agarose-based skin biological phantoms that mimic the melanoma tumoral tissue and surrounding healthy tissue, demonstrating the preservation of the photothermal properties of the AuNTs after their incorporation in the phantoms. By employing two different methods, fluorescence staining and MTT assay, we also demonstrated the in vitro therapeutic effect of the proposed system, specifically that AuNTs@780 exhibit the highest phototoxic effect on B16.F10 melanoma cells after NIR laser irradiation.

Therefore, we can confidently conclude that AuNTs@780 with LSPR band in-resonance with 785 nm laser used to induce the PTT effect, are efficient PTT agents with a high photothermal conversion efficacy. In further clinical studies, these results allow us to reduce the required laser power density and irradiation time to reach a high enough temperature able to induce an efficient PTT effect in vivo.

## Supplementary Materials

The following supporting information can be downloaded at: https://www.mdpi.com/article/10.3390/ijms232213724/s1.

## References

[1] Smith S., Prewett S. (2020). Principles of chemotherapy and radiotherapy. Obstet. Gynaecol. Reprod. Med..

[2] Yazbeck V., Alesi E., Myers J., Hackney M.H., Cuttino L., Gewirtz D.A. (2022). An overview of chemotoxicity and radiation toxicity in cancer therapy. Advances in Cancer Research.

[3] Kumar A.V.P., Dubey S.K., Tiwari S., Puri A., Hejmady S., Gorain B., Kesharwani P. (2021). Recent advances in nanoparticles mediated photothermal therapy induced tumor regression. Int. J. Pharm..

[4] Kadkhoda J., Tarighatnia A., Barar J., Aghanejad A., Davaran S. (2022). Recent advances and trends in nanoparticles based photothermal and photodynamic therapy. Photodiagnosis Photodyn. Ther..

[5] Zhi D., Yang T., O’Hagan J., Zhang S., Donnelly R.F. (2020). Photothermal therapy. J. Control. Release.

[6] Ali M.R.K., Wu Y., El-Sayed M.A. (2019). Gold-Nanoparticle-Assisted Plasmonic Photothermal Therapy Advances Toward Clinical Application. J. Phys. Chem. C.

[7] Taylor M.L., Wilson R.E., Amrhein K.D., Huang X. (2022). Gold Nanorod-Assisted Photothermal Therapy and Improvement Strategies. Bioengineering.

[8] Yi X., Duan Q.-Y., Wu F.-G. (2021). Low-Temperature Photothermal Therapy: Strategies and Applications. Research.

[9] Bao Z., Liu X., Liu Y., Liu H., Zhao K. (2016). Near-infrared light-responsive inorganic nanomaterials for photothermal therapy. Asian J. Pharm. Sci..

[10] Abadeer N.S., Murphy C.J. (2016). Recent Progress in Cancer Thermal Therapy Using Gold Nanoparticles. J. Phys. Chem. C.

[11] Rau L.-R., Huang W.-Y., Liaw J.-W., Tsai S.-W. (2016). Photothermal effects of laser-activated surface plasmonic gold nanoparticles on the apoptosis and osteogenesis of osteoblast-like cells. Int. J. Nanomed..

[12] Krajnik B., Golacki L.W., Fiedorczyk E., Bański M., Noculak A., Holodnik K.M., Podhorodecki A. (2021). Quantitative comparison of luminescence probes for biomedical applications. Methods Appl. Fluoresc..

[13] Ma X., Cheng Y., Huang Y., Tian Y., Wang S., Chen Y. (2015). PEGylated gold nanoprisms for photothermal therapy at low laser power density. RSC Adv..

[14] Kim M., Lee J.-H., Nam J.-M. (2019). Plasmonic Photothermal Nanoparticles for Biomedical Applications. Adv. Sci..

[15] Yang W., Xia B., Wang L., Ma S., Liang H., Wang D., Huang J. (2021). Shape effects of gold nanoparticles in photothermal cancer therapy. Mater. Today Sustain..

[16] Vines J.B., Yoon J.-H., Ryu N.-E., Lim D.-J., Park H. (2019). Gold Nanoparticles for Photothermal Cancer Therapy. Front. Chem..

[17] Maestro L.M., Haro-González P., Sánchez-Iglesias A., Liz-Marzán L.M., García Solé J., Jaque D. (2014). Quantum Dot Thermometry Evaluation of Geometry Dependent Heating Efficiency in Gold Nanoparticles. Langmuir.

[18] Wang Y., Black K.C.L., Luehmann H., Li W., Zhang Y., Cai X., Wan D., Liu S.-Y., Li M., Kim P. (2013). Comparison Study of Gold Nanohexapods, Nanorods, and Nanocages for Photothermal Cancer Treatment. ACS Nano.

[19] Gao Y., Li Y., Wang Y., Chen Y., Gu J., Zhao W., Ding J., Shi J. (2015). Controlled Synthesis of Multilayered Gold Nanoshells for Enhanced Photothermal Therapy and SERS Detection. Small.

[20] Knights O., Freear S., McLaughlan J.R. (2020). Improving Plasmonic Photothermal Therapy of Lung Cancer Cells with Anti-EGFR Targeted Gold Nanorods. Nanomaterials.

[21] Ren Y., Chen Q., Qi H., Ruan L. (2017). Experimental Comparison of Photothermal Conversion Efficiency of Gold Nanotriangle and Nanorod in Laser Induced Thermal Therapy. Nanomaterials.

[22] Boca S.C., Potara M., Gabudean A.-M., Juhem A., Baldeck P.L., Astilean S. (2011). Chitosan-coated triangular silver nanoparticles as a novel class of biocompatible, highly effective photothermal transducers for in vitro cancer cell therapy. Cancer Lett..

[23] Maturi M., Locatelli E., Sambri L., Tortorella S., Šturm S., Kostevšek N., Comes Franchini M. (2021). Synthesis of Ultrasmall Single-Crystal Gold-Silver Alloy Nanotriangles and Their Application in Photothermal Therapy. Nanomater. Basel Switz..

[24] Suarasan S., Focsan M., Soritau O., Maniu D., Astilean S. (2015). One-pot, green synthesis of gold nanoparticles by gelatin and investigation of their biological effects on Osteoblast cells. Colloids Surf. B Biointerfaces.

[25] Lai W.-F., Wong W.-T. (2021). Property-Tuneable Microgels Fabricated by Using Flow-Focusing Microfluidic Geometry for Bioactive Agent Delivery. Pharmaceutics.

[26] Khodashenas B., Ardjmand M., Rad A.S., Esfahani M.R. (2021). Gelatin-coated gold nanoparticles as an effective pH-sensitive methotrexate drug delivery system for breast cancer treatment. Mater. Today Chem..

[27] Lai W.-F. (2021). Development of Hydrogels with Self-Healing Properties for Delivery of Bioactive Agents. Mol. Pharm..

[28] Obireddy S.R., Lai W.-F. (2021). Multi-Component Hydrogel Beads Incorporated with Reduced Graphene Oxide for pH-Responsive and Controlled Co-Delivery of Multiple Agents. Pharmaceutics.

[29] Fernandes D.A., Fernandes D.D., Malik A., Gomes G.-N.W., Appak-Baskoy S., Berndl E., Gradinaru C.C., Kolios M.C. (2021). Multifunctional nanoparticles as theranostic agents for therapy and imaging of breast cancer. J. Photochem. Photobiol. B.

[30] Sakthi Devi R., Girigoswami A., Siddharth M., Girigoswami K. (2022). Applications of Gold and Silver Nanoparticles in Theranostics. Appl. Biochem. Biotechnol..

[31] Barani M., Rahdar A., Mukhtar M., Razzaq S., Qindeel M., Hosseini Olam S.A., Paiva-Santos A.C., Ajalli N., Sargazi S., Balakrishnan D. (2022). Recent application of cobalt ferrite nanoparticles as a theranostic agent. Mater. Today Chem..

[32] Campu A., Craciun A.-M., Focsan M., Astilean S. (2019). Assessment of the photothermal conversion efficiencies of tunable gold bipyramids under irradiation by two laser lines in a NIR biological window. Nanotechnology.

[33] Vardaki M.Z., Kourkoumelis N. (2020). Tissue Phantoms for Biomedical Applications in Raman Spectroscopy: A Review. Biomed. Eng. Comput. Biol..

[34] Kim M., Kim G., Kim D., Yoo J., Kim D.-K., Kim H. (2019). Numerical Study on Effective Conditions for the Induction of Apoptotic Temperatures for Various Tumor Aspect Ratios Using a Single Continuous-Wave Laser in Photothermal Therapy Using Gold Nanorods. Cancers.

[35] Gupta N., Malviya R. (2021). Understanding and advancement in gold nanoparticle targeted photothermal therapy of cancer. Biochim. Biophys. Acta BBA–Rev. Cancer.

[36] Campu A., Focsan M., Lerouge F., Borlan R., Tie L., Rugina D., Astilean S. (2020). ICG-loaded gold nano-bipyramids with NIR activatable dual PTT-PDT therapeutic potential in melanoma cells. Colloids Surf. B Biointerfaces.

[37] Zhu X., Feng W., Chang J., Tan Y.-W., Li J., Chen M., Sun Y., Li F. (2016). Temperature-feedback upconversion nanocomposite for accurate photothermal therapy at facile temperature. Nat. Commun..

[38] Nagy-Simon T., Potara M., Craciun A.-M., Licarete E., Astilean S. (2018). IR780-dye loaded gold nanoparticles as new near infrared activatable nanotheranostic agents for simultaneous photodynamic and photothermal therapy and intracellular tracking by surface enhanced resonant Raman scattering imaging. J. Colloid Interface Sci..

[39] Suarasan S., Craciun A.-M., Licarete E., Focsan M., Magyari K., Astilean S. (2019). Intracellular Dynamic Disentangling of Doxorubicin Release from Luminescent Nanogold Carriers by Fluorescence Lifetime Imaging Microscopy (FLIM) under Two-Photon Excitation. ACS Appl. Mater. Interfaces.

[40] Mustari A., Nishidate I., Wares M.A., Maeda T., Kawauchi S., Sato S., Sato M., Aizu Y. (2018). Agarose-based Tissue Mimicking Optical Phantoms for Diffuse Reflectance Spectroscopy. J. Vis. Exp. JoVE.

[41] Nishidate I., Tanaka N., Kawase T., Maeda T., Yuasa T., Aizu Y., Yuasa T., Niizeki K. (2011). Noninvasive imaging of human skin hemodynamics using a digital red-green-blue camera. J. Biomed. Opt..

[42] Câmpu A.-M. (2020). Implementation of Gold Nano-bipyramids as Efficient Biosensing Enhancers and Thermo-plasmonic Generators. Ph.D. Thesis.

