# Supplementary material for: Assessing the Efficiency of Triangular Gold Nanoparticles as NIR Photothermal Agents In Vitro and Melanoma Tumor Model
Source: Int J Mol Sci. 2022 Nov 8;23(22):13724. doi: 10.3390/ijms232213724 (PMC9695152; doi:10.3390/ijms232213724)
Supplement: Supplementary file 1 [file ijms-23-13724-s001.zip › ijms-1964134-supplementary.pdf]

Supplementary Materials

Assessing the efficiency of triangular gold nanoparticles as NIR photothermal agents *in vitro* and melanoma tumor model

Sorina Suarasan[a]*, Andreea Campu[a], Adriana Vulpoi[b], Manuela Banciu[c], and Simion Astilean[a,d]

[a]Nanobiophotonics and Laser Microspectroscopy Center, Interdisciplinary Research Institute in Bio-Nano-Sciences, Babes-Bolyai University, T. Laurian Str. 42, 400271 Cluj-Napoca, Romania

[b] Nanostructured Materials and Bio-Nano-Interfaces Center, Interdisciplinary Research Institute in Bio-Nano-Sciences, Babes-Bolyai University, T. Laurian 42, 400271, Cluj-Napoca, Romania

[c]Department of Molecular Biology and Biotechnology, Center of Systems Biology, Biodiversity and Bioresources, Faculty of Biology and Geology, Babes-Bolyai University, Cluj-Napoca, Romania

[d]Department of Biomolecular Physics, Faculty of Physics, Babes-Bolyai University, M Kogalniceanu Str. 1, 400084 Cluj-Napoca, Romania

*Corresponding author: sorina.suarasan@ubbcluj.ro

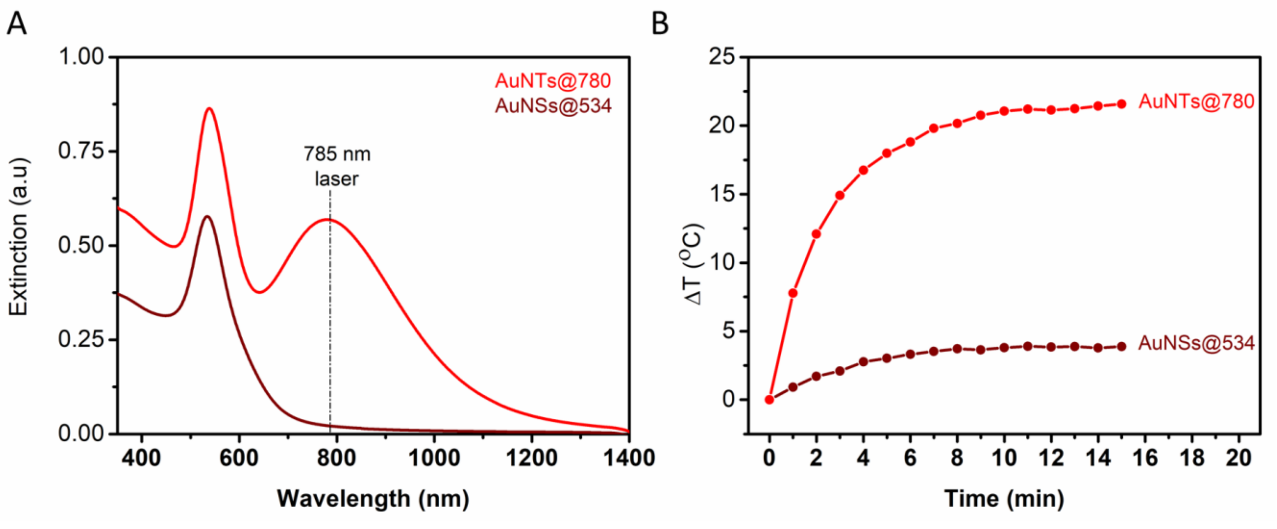

Figure S1. A. LSPR spectra of AuNTs@780 compared to AuNSs@534. **B.** The thermal curves recorded from AuNTs@780 and AuNSs irradiated by a 785 nm laser for 15 min

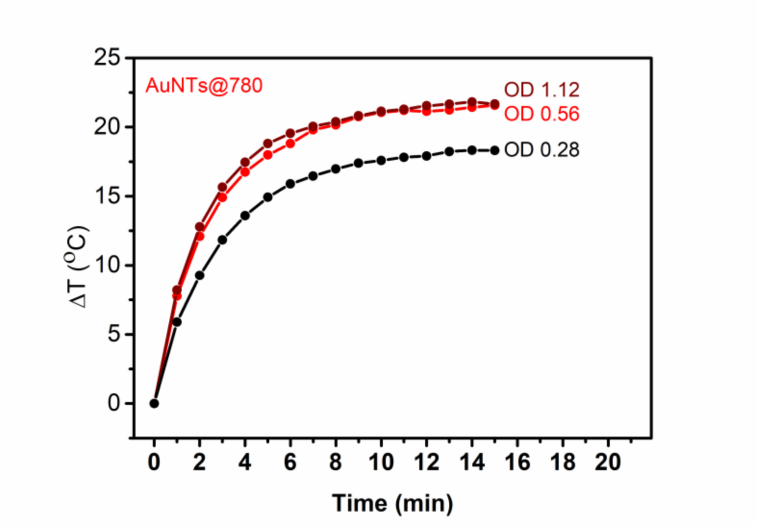

Figure S2. The AuNTs@780 thermal curves generated with regard to the variation of the optical density when irradiated in the same experimental conditions.

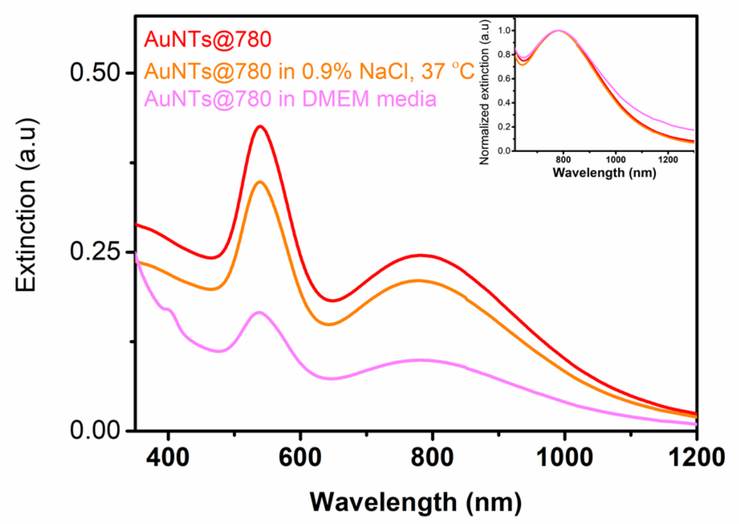

Figure S3. AuNTs stability in physiological conditions

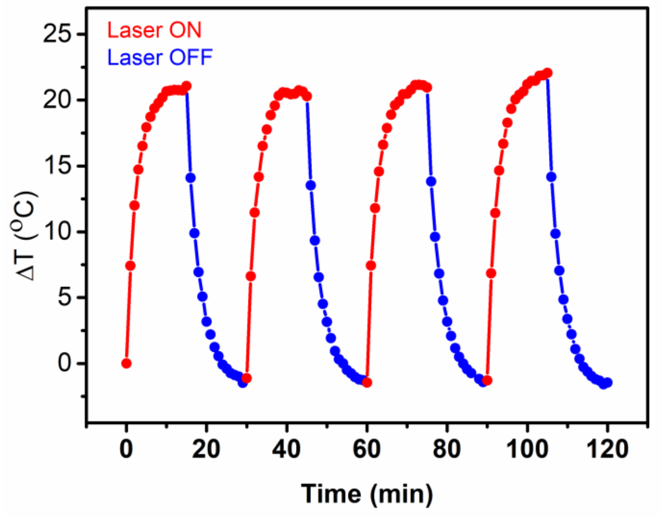

Figure S4. The stability of AuNTs inside biological phantoms after 4 NIR laser ON-OFF cycles.

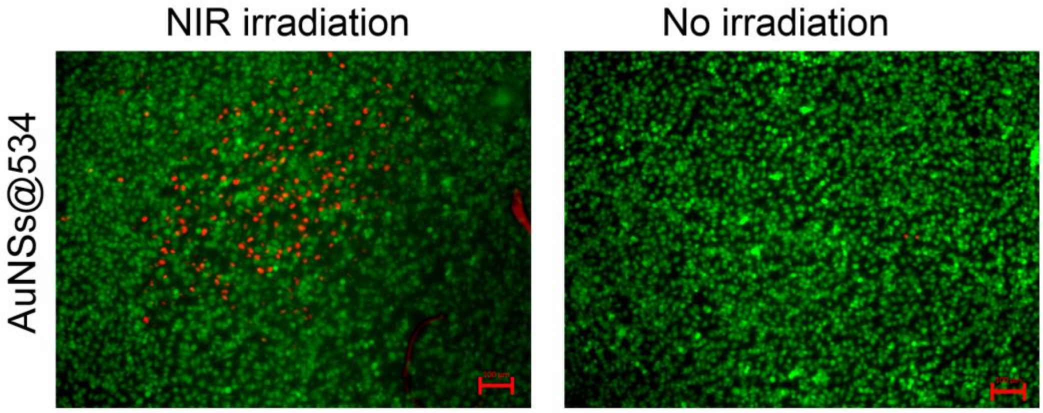

Figure S5. Merged fluorescence images of B16.F10 cells stained with calcein-AM and PI before and after NIR irradiation
